# What Is Health?

**Published:** 1862-05

**Authors:** 


**WHAT IS HEALTH?**—"*Mens sana, in corpore sano*" is the most philosophical and most comprehensive definition of health ever given—a sound mind in a sound body. It has been recognized as a truth ever since it was first uttered, seventeen hundred years ago, by the satirical poet Juvenal; and as if he were to demonstrate in his own person the correctness of his definition, he died of mortification in his eighty-first year — died of a mental malady. Physiologists have generally settled down on the fact that the mind of itself is always sound, is never originally diseased; that it becomes so in every single case, in consequence of its connection with the body; that no form of insanity can exist except as caused by a diseased condition of some part of the corporeal frame. Such being the case, it is the dictate of wisdom, it is the paramount duty of man to study how continuous and vigorous health may be secured and preserved to a serene old age.

